# Spatial Learning in Naturalistic Search With Simulated Vision Loss

**DOI:** 10.1167/tvst.15.4.10

**Published:** 2026-04-14

**Authors:** Kirsten Veerkamp, Daniel Müller, Christian N. L. Olivers, David L. Mann

**Affiliations:** 1 Amsterdam Movement Sciences & Institute for Brain and Behavior Amsterdam, Department of Human Movement Sciences, Vrije Universiteit Amsterdam, Amsterdam, the Netherlands; 2 Institute for Brain and Behavior Amsterdam, Department of Experimental and Applied Psychology, Vrije Universiteit Amsterdam, Amsterdam, the Netherlands

**Keywords:** visual search, virtual reality, low vision, visual field loss, low vision navigation, spatial memory

## Abstract

**Purpose:**

In everyday life, we learn to find relevant objects in complex three-dimensional environments. This spatial learning is even more important under conditions of visual impairment. Yet, little is known about how spatial learning and impaired vision interact to shape search behavior in complex naturalistic settings. Here we assessed how spatial learning is used to mitigate the consequences of acute (simulated) central and peripheral vision loss for navigating an everyday three-dimensional environment.

**Methods:**

Seventy-five participants were assigned to one of three simulated vision conditions (full vision, central mask, or peripheral mask) and performed multiple searches for products in a virtual reality supermarket.

**Results:**

Task completion times and navigational efficiency were affected by reduced vision but improved substantially for repeated product searches. Improvements were more pronounced with simulated vision loss, especially with simulated peripheral loss. Gaze and other orienting behavior also changed with learning, leading to more scanning at the initiation of the search under peripheral loss, and less scanning during the actual search, particularly with central loss.

**Conclusions:**

Spatial learning affects visual orienting behavior and aids in compensating for the detrimental consequences of vision loss in everyday search behavior.

**Translational Relevance:**

These results emphasize the importance of spatial consistencies in dealing with visual impairments in everyday environments.

## Introduction

Daily life is filled with visual searches for objects that we need or want, such as finding the coffee in the supermarket. Unlike in typical lab studies of visual search, in naturalistic real-life settings spatial memory plays an important role, as we become familiar with complex environmental layouts. For example, finding the coffee in our regular supermarket is faster than when finding it in a store never visited before. Spatial learning probably becomes even more important when vision is impaired, because of the reduced availability of real-time visual information. Here we address how spatial learning interacts with central and peripheral vision impairment in shaping visual search behavior.

Several studies have examined spatial learning in naturalistic real-world or virtual reality (VR) settings under conditions of normal vision.^1^^–^^4^ A handful of studies have investigated spatial learning under conditions of impaired vision in naturalistic environments, but none have yet directly compared the impact of central and peripheral vision loss.^5^^–^^7^ In the study by Rand et al.,^7^ normally sighted participants navigated through a university building with normal vision or while wearing blurring goggles causing reduced visual acuity. Although participants walked through the environment, the experimenter pointed out certain landmarks along the route. Afterward, participants were asked to recall where specific landmarks were located. Pointing errors were larger when participants had walked with blurred vision than with normal vision, indicating poorer spatial memory. However, the overall blurred vision meant that both central and peripheral vision were affected, and thus the influence of each on spatial learning could not be compared. Using a similar paradigm, Barhorst-Cates et al.^5^^,^^6^ focused on the effect of specifically peripheral vision loss on spatial learning. In their studies, normally sighted participants walked either through a hallway or an art museum while wearing goggles that either did or did not restrict their visual field to a central aperture. Spatial learning was worse with the peripheral vision loss, as reflected by a poorer recall of the locations of items seen along the path. There was no central vision loss condition, plus the central aperture was tied to the glasses and not the eyes, forcing people to make head movements to be able to foveate areas of interest. This factor may obscure changes in eye movement patterns that might occur under more natural viewing conditions. In another study, Beitner et al.^13^ investigated spatial memory for a VR scene under full lighting conditions vs. when the scene was dark and could only be inspected through a flashlight-contingent window. The latter could be regarded as mimicking having only central vision, on the assumption that the eyes would follow the light (which is not a given). They observed no differences in spatial learning, which implies that foveal vision was sufficient for learning their environment. Finally, some studies have directly compared effects of simulated central and peripheral vision loss on naturalistic search behavior in VR,^8^^,^^9^^,^^11^ but these have not assessed spatial learning. Insights could help rehabilitation specialists to aid those with vision loss, particularly in the early phases of loss before becoming well-accustomed to their impairment.

The aim of our study was to assess the degree to which spatial learning mitigates the consequences of central and peripheral vision loss in a naturalistic search task. To do so, we analyzed behavior while participants performed a visual search task in a VR supermarket environment under one of three vision conditions: full vision, central mask, or peripheral mask. We tracked task performance as well as body, head, and eye movements. Within the task, participants performed a series of both novel and repeated searches to examine the role of spatial learning in search. We hypothesized that task performance would improve in repeated searches in all vision conditions, as reflected by faster task completion times and more efficient navigation in repeated searches (cf. Kit et al. and Li et al.).^1^^–^^3^ Furthermore, because the search was expected to become more targeted with learning, we expected repetitions to be accompanied by fewer head and eye rotations in all conditions (cf. Li et al.).^3^ Of most interest was how spatial learning would interact with the simulated vision impairment. Here our expectations were less defined. Following the aforementioned studies by Barhorst-Cates et al. and Rand et al.,^5^^–^^7^ it seemed reasonable to hypothesize that learning would be worse in the presence of impaired vision. Alternatively, initial search performance is likely to be worse to start with in the presence of vision loss, and therefore participants might actually benefit relatively more from spatial learning in a naturalistic environment given the contextually and structurally rich cues and a lower level of initial performance. If so, benefits with repetition would be greater with peripheral vision loss than with central vision loss, because search performance with peripheral loss has been found to be worse to begin with.^8^^,^^9^ To foreshadow, we observed evidence for the latter. We then explored further what led to these performance changes by investigating underlying orienting behavior.

## Methods

The current study is based on a dataset from a study previously described in Veerkamp et al.^9^ The previous study focused on the effects of vision loss on overall search behavior and did not address spatial learning. The analyses presented here are new.

### Participants

A total of 84 participants took part in exchange for course credits or money. The protocol was approved by the Scientific and Ethics Review Board of the Faculty of Behaviour and Movement Sciences at the Vrije Universiteit Amsterdam, and the research was performed in accordance with the Declaration of Helsinki. Informed consent was obtained from all participants. Exclusion criteria were color vision deficiency, flash-induced epilepsy, and known VR-related motion sickness. Each participant had normal or corrected-to-normal vision (10 wore glasses, 18 wore contact lenses). Nine participants were excluded due to either a technical issue (*n* = 6), a low percentage of valid gaze data (*n* = 2), or not being familiar with a target product that they were required to search for in our environment (*n* = 1). Hence, a sample of 75 participants remained (mean age, 21 years; range, 18–30 years; 52 females), of which 25 were assigned to each of the three simulated vision conditions: full vision, central mask (central vision loss), or peripheral mask (peripheral vision loss).

### VR Environment

#### Virtual Supermarket

**Figure 1.**
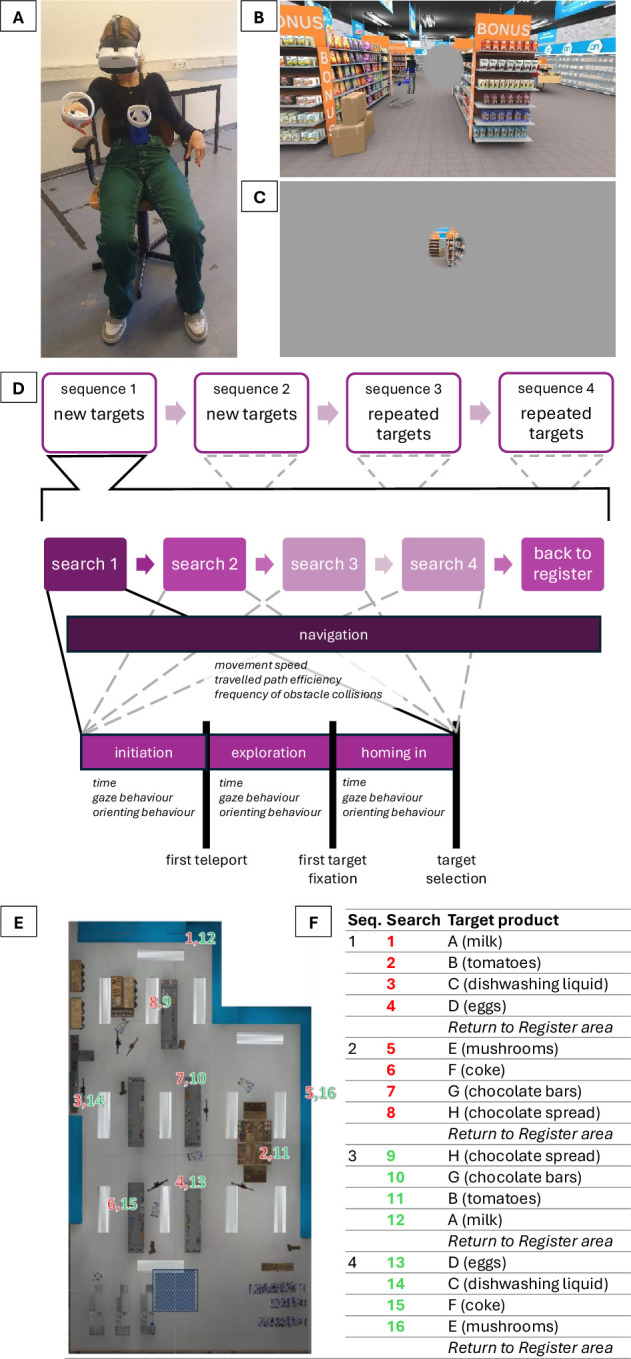
(**A**) A participant while performing the experiment. (**B**) The VR supermarket environment seen from the starting position while central vision loss is simulated by a central mask. (**C**) The VR supermarket environment while peripheral vision loss is simulated by a peripheral mask. (**D**) An overview of the experiment consisting of four search sequences, with four target products in each. The outcome measures obtained from the different phases for each product search are shown. (**E**) An aerial view of the virtual supermarket. Each number indicates the location of the target product of the respective search. In red, new target searches are displayed (searches 1–8), and in green, repeated target searches are displayed (searches 9–16). Note that the second searches started from different starting points than the first searches. The checkered plane indicates the start and end location of each sequence. (**F**) An overview of all four search sequences.

The virtual supermarket was developed in Unity (Unity Technologies, USA; version 2023.2.1f1) and resembled a local supermarket from a popular Dutch chain (Fig. 1A). We modified the VirtuMart as developed and kindly provided by van der Laan et al.^10^ We added more products, resulting in a total of 719 different products, with multiple instances of a product typically being grouped together on a shelf. After arranging the products in a structured way, small, randomized deviations in their position and rotation were added to each individual product to increase realism. Shelves were placed following a typical supermarket layout incorporating areas and aisles with certain themes (e.g., fruit and vegetables, bread, soft drinks, dairy products, and cleaning products). The two ends of each aisle contained a variety of products on discount (i.e., special offer), not linked to the aisle's theme, and that were thus placed out of context (although still consistent with the general context of a supermarket). Trolleys, boxes, and avatars pivoting around their position were placed more or less randomly around the supermarket to increase realism.

A Pico Neo 3 Pro Eye VR headset (Pico Interactive, Beijing, China) with integrated Tobii eye tracking (120 Hz; VR4 Platform; Tobii Technology, Stockholm, Sweden) was used to display the virtual environment. The headset has a binocular field of view of 98°. Participants were seated on a swivel chair while wearing the headset and holding one controller (Fig. 1A). Locomotion was provided by means of stepwise teleportation to prevent motion sickness. Pressing the trigger button on the back of the controller resulted in moving 0.5 m in the horizontal plane in the virtual environment in the direction in which the controller was pointing. If the movement caused the participant to collide with an object, the participant was moved 0.5 m backward, and haptic feedback was provided by a controller vibration (note that the supermarket was too large to have participants walk around in real space; furthermore, we planned to use the same task and environment with elderly or otherwise less mobile people). A product could be selected by holding the controller within 0.5 m of the product, while pressing and holding the trigger button on the controller for 3 seconds. Another controller was strapped to the chest of the participant to register body rotation independent of head rotation.

#### Test Protocol

The test protocol consisted of three steps, as in Veerkamp et al.^9^: calibration, familiarization, and testing (which itself consisted of four search sequences). First, the eye tracker was calibrated using a custom calibration created with Tobii Ocumen SDK to make it suitable for testing people with and without vision impairment. The background color was set to black, and the moving calibration stimulus consisted of a large gray dot and a large bright X spanning the entire screen. Calibration was deemed successful according to the standard Tobii algorithm criteria. A calibration check was performed using five points before each search sequence. In case of failure, or if the headset had been taken off in the meantime, the calibration was repeated. During the experiment, the participant's view was livestreamed from the headset to a laptop to check that the mask was responding as expected.

Next, participants were made familiar with the controls to navigate the VR supermarket using a layout different from that used in the experiment proper, and with empty shelves. In this environment, participants were free to explore and become familiar with the means of locomotion and for selecting products. To this end, we also gave participants the assignment to find a checkered ball somewhere in the empty supermarket. Furthermore, participants were shown one shelf containing only the target products that they would be required to search for in the experiment proper to allow participants to become familiar with the products (but not where they would be).

During the experiment itself, participants performed four search sequences in the virtual supermarket. The first and second sequences consisted of searches for new targets, and the third and fourth sequences consisted of searches for those earlier searched-for targets. When participants searched for a product the second time, both the product and its location were repeated, but the order in which they searched for those eight products was different (see Fig. 1E). This meant that the starting point from where participants searched for those products was different the second time they looked for them, because it depended on the location of the previous target product. At the start of each sequence, participants were instructed to search for four target products in the order specified on the shopping list (so 16 product searches in total; see Fig. 1F for an overview). Each participant received the same shopping list to search for the same eight products. We selected the eight target products so that they were from different categories that were distributed throughout the supermarket (Fig. 1E) and placed at different heights (although not the highest and lowest shelves, to ensure visibility), with all products considered to be commonly known products among the wider population. Familiarity with the products was confirmed with each participant in the familiarization trial. The shopping list was provided by means of a verbal recording at the beginning of each sequence and could be repeated by pressing a button on the controller. The list only included the four search targets for that specific search sequence. Participants were encouraged to memorize the shopping list. However, if needed, the shopping list could be recalled by pressing the same button during the search. All products remained in the same location for all participants throughout the experiment. Each sequence started at the supermarket entrance and ended with participants moving to the checkout register area. In between, participants searched for the designated products in order. An individual product search ended only when the correct target product was selected. If the wrong product was selected, auditory feedback was provided and the search continued. We a priori adopted the rule that if an individual product search took longer than 5 minutes, then the participant would be guided toward the correct product by the experimenter. However, this did not occur. Each sequence consisted of one product that was on discount and therefore not in its usual place (i.e., placed out of context). We did not further analyze the effect of this factor, because participants were instructed that the target was placed in one of the discount areas, which were clearly demarcated areas at the front end of aisles. Therefore, these products were relatively easy to find and so it did not turn out to be a particularly useful manipulation of context. Also, one-half of the searches were performed at normal (high) contrast, and one-half at low contrast as imposed by means of a smoky mask (RGBA: [48 48 48 100]) displayed in front of the camera. Each product occurred in both a low- and a high-contrast search, and the number of low- and high-contrast searches was matched between the first and second halves of the experiment. Contrast effects were not analyzed in the current study, because they have been addressed previously, when we found that contrast mostly challenged search with peripheral vision loss (for further details see Veerkamp et al.).^9^ The supermarket layout and product placement remained the same throughout the four sequences. Illustrative screen recordings of one of the authors conducting the first search sequence under each of the vision conditions are provided in the Supplementary Material.

### Vision Loss Simulations

Vision loss was simulated for each eye separately using a gaze-contingent mask. Central vision loss was modelled as a circular mask of 6° in front of each eye (Fig. 1C). Peripheral vision loss was modelled as a mask with a 6° circular aperture (Fig. 1D). For both simulations the edge was softened with a 1.5° linear fade. The size of the 6° mask or window was consistent with what has been used in previous VR studies as a suitable trade-off between central and peripheral vision loss.^8^^,^^11^ Moreover, approximately 50% of the visual cortex is devoted to the central 6° of the visual field, and the other 50% covers the remaining peripheral part of the visual field,^12^ resulting in an equal distribution of cortical coverage in our case.

A limitation of gaze-contingent simulations is a potential delay between when gaze changes and when the gaze-contingent change follows. Although we did everything to keep the delay as short as possible, we had no means to estimate its exact value. Also, owing to some complexities in the VR computations that we later simplified, the frame rate was 23 Hz on average for the first 35 participants, and it was 67 Hz for the remaining 40 participants. This probably also affected the gaze-contingent delay. Importantly, participants with the lower frame rate were evenly distributed across conditions (full vision: *n* = 12; central mask: *n* = 12; peripheral mask: *n* = 11). Furthermore, we have confirmed that the difference in frame rate did not affect our conclusions either descriptively or when including those participants as separate groups in our statistical analyses (see Results and the Supplementary Material). In the Results of this paper, we indicate the data points for participants tested with the lower frame rate by using a lighter shade.

### Data Analysis and Statistics

Spatial learning was assessed by comparing new target searches (sequences 1 and 2) to repeated target searches (sequences 3 and 4). We first compared task performance, after which we assessed the various behavioral components that might result in potential differences in task performance. Data analyses and statistics were performed in MATLAB 2024b (The MathWorks, Natick, MA). Outcomes were calculated as in Veerkamp et al.,^9^ but instead of aggregating outcomes across all four search sequences, they were now analyzed separately for new target searches (sequences 1 and 2) and for repeated target searches (sequences 3 and 4) to assess spatial learning.

Task performance was assessed by measuring the completion time and navigation efficiency for each product search independently. The completion time (in seconds) for each search was defined as the time elapsed from the selection of the previous target to the moment the participant selected the search target. The search time for the first search of each sequence started with the first teleport after hearing the shopping list. Navigation efficiency was assessed for each search by quantifying the movement speed, travelled path efficiency, and frequency of obstacle collisions. Movement speed (in m/s) was calculated by summing the Euclidean distance between body three-dimensional (3D) samples and dividing it by the search completion time. Note that a higher movement speed reflects more frequent teleports. Travelled path efficiency was calculated by dividing the length of the travelled path by the shortest path possible. The shortest path was obtained by exporting the supermarket's navigation mesh and target product center positions from Unity, and using an A* algorithm in MATLAB. The frequency of obstacle collisions was defined as the number of collisions with trolleys or boxes in the supermarket, divided by the travelled distance (i.e., collisions per meter).

Following Veerkamp et al.,^9^ duration, gaze, and other orienting behavior were analyzed for the separate search phases (initiation, exploration and homing-in phases). The duration of the search initiation phase was defined by the period from the moment the shopping list was presented (first search of each sequence), or the moment the previous product was found (searches 2–4), until the first teleport was made. The exploration phase spanned the moment from the first teleport until the first fixation on the target product. The homing-in phase spanned from the first target fixation until the target product was selected using a correct button press. To analyze gaze behavior, eye (eye in head) and gaze (i.e., head + eye in world) 3D direction vectors were converted to degrees. The angular change between consecutive direction vectors was then divided by the difference in time between these two vectors to obtain the velocity over time. The velocity signal was smoothened by a second-order Savitzky–Golay filter with a frame length of five. Fixations were identified as gaze changes below 3° or below 30°/s in a time window of 80 ms or longer. All other samples were classified as saccades. Fixation rate (i.e., number of fixations per second) and fixation duration (in milliseconds) were obtained from the gaze data; saccade amplitude (in degrees) was obtained from the eye data. Other orienting behaviors were quantified by converting the 3D body and head direction unit vectors into angular changes over time, summing these rotation timeseries data over time, and dividing this summed rotation by the search completion time. This approach resulted in body rotation normalized for time and head rotation normalized for time (each in deg/s).

For all outcomes, outliers were identified at the participant level as values less than the first quartile minus 1.5 times the interquartile range, or greater than the third quartile plus 1.5 times the interquartile range. These outliers were excluded from the statistical analyses, but displayed and indicated in the figures for transparency.

Outcomes were calculated and averaged across the target searches for the eight new products (sequences 1 and 2) and for the repeated target searches for those eight products (sequences 3 and 4). A repeated measures 3 × 2 analysis of variance was performed with target repetition (new, repeated) and vision condition (full vision, central mask, peripheral mask) as factors. If there was a significant interaction effect of target repetition and vision condition, two types of post hoc tests were performed. Paired *t* tests between new and repeated searches for each vision condition provided insights into which vision conditions were affected by target repetition. Additionally, repeated measures 2 × 2 analyses of variance between each pair of vision conditions, and their interaction effect, allowed us to better understand which of the vision conditions was affected more by target repetition. For each of the post hoc tests, *P* values were Bonferroni corrected for the number of comparisons (three in each). Similarly, outcomes were compared between sequences 1 and 2 to assess spatial learning for new targets within the same environment. Again, overall condition effects were not assessed here, because they have been described previously in Veerkamp et al.^9^ We do not present results at a product-based level given that the starting point of the search differed between the first and second search for each product.

## Results

The general effects of the simulated vision loss on performance have been extensively reported in Veerkamp et al.^9^ To summarize, vision loss led to slower and less accurate searches, including less efficient navigation through the virtual supermarket, as well as less efficient homing in on the target—with performance especially affected by peripheral vision loss. Furthermore, central vision loss led to more and shorter fixations, whereas peripheral vision loss resulted in smaller saccades. Here we focus on the learning effects and how these interact with vision loss.

We first report overall task performance averaged across all eight target products for new and repeated target searches (comparing sequences 1 and 2 with sequences 3 and 4), for each vision condition. We then explore the underlying factors that do and do not contribute to differences in overall task performance as a function of learning, including navigation, gaze, and orienting behavior, for each of the three phases of search (i.e., the initiation, exploration, and homing-in phases). A table containing an overview of all outcome variables (e.g., means, standard deviations, and *P* values) is provided in the Supplementary Material A. In the graphs we indicate the low frame rate group (lighter shades) and high frame rate group (darker shades; see Methods for why this differed). Unless mentioned, none of the conclusions were affected by frame rate (as assessed through additional analyses with frame rate as a between-participants factor). If anything, the differences between vision conditions were more pronounced for the high frame rate group.

### General Task Performance as a Function of Learning

Participants rarely selected the wrong product (2.6% of product searches). Given the low number of errors, it was not informative to perform comparisons of the error rate between new and repeated searches or across vision conditions. Trials in which the wrong product was selected were excluded from further analysis.

#### Task Completion Time

**Figure 2.**
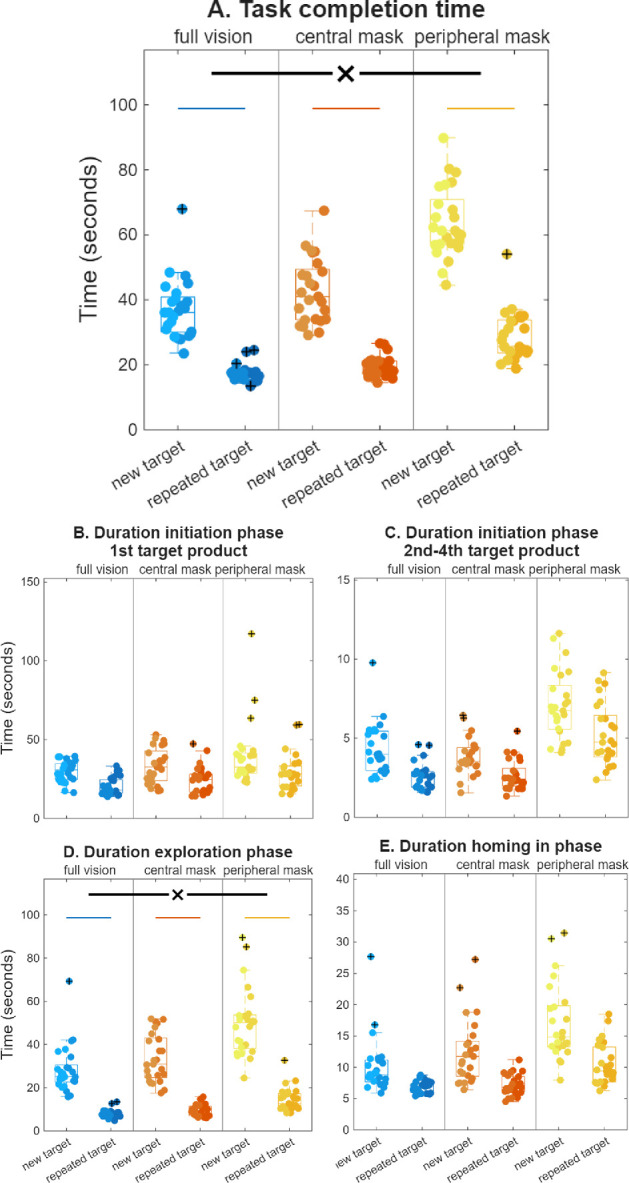
(**A**) Task completion time for new and repeated target searches across the three vision simulation conditions. (**B**) The duration of the initiation phase for the first target product of each sequence, in which the shopping list was memorized before starting the search. (**C**) The time to start the search for the second, third and fourth target product of each sequence. (**D**) The duration of the exploration phase, which lasted from the first teleport up to the first fixation on the target product. (**E**) The duration of the homing-in phase, which lasted from the first fixation on the target product until the target was selected. A black *x* with horizontal lines indicates a significant interaction effect between new and repeated target searches across the different vision conditions. Horizontal colored lines indicate there was a significant difference between new and repeated searches for the corresponding vision condition. Boxplots are displayed for each condition, with the central line indicating the median, the edges indicating the 25th and 75th percentile, and the whiskers extending to the minimum and maximum values not considered to be outliers. Each dot represents the average across searches from an individual participant, black + signs indicate outliers. Lighter shade indicates participants who were tested with the lower frame rate.

Across all vison conditions, the completion time was significantly shorter for the repeated searches, *F*(1, 66) = 584.33, *MSE* = 23274.71, *P* < 0*.*001 (Fig. 2A). The interaction effect between target repetition and vision condition was significant, *F*(2, 66) = 20.19, *MSE* = 804.17, *P* < 0*.*001. Although repetition resulted in benefits in all conditions, full vision: Δ = −19.4 ± 6.8 s, *t*(19) = 12.70, *P* < 0*.*001; central mask: Δ = −23.4 ± 9.5 s, *t*(24) = 12.28, *P* < 0*.*001; peripheral mask: Δ = −35.5 ± 9.8 s, t(23) = 17.81, *P* < 0*.*001, these benefits were significantly larger in the peripheral mask condition than in the full vision condition, *F*(1, 42) = 38.88, *MSE* = 1425.54, *P* < 0*.*001, and central mask condition, *F*(1, 47) = 19.38, *MSE* = 901.90, *P* < 0*.*001. The repetition benefit did not differ significantly between the full vision and central mask conditions, *F*(1,43) = 2.53, *MSE* = 90.19, *P* = 0.36.

#### Navigation Efficiency

**Figure 3.**
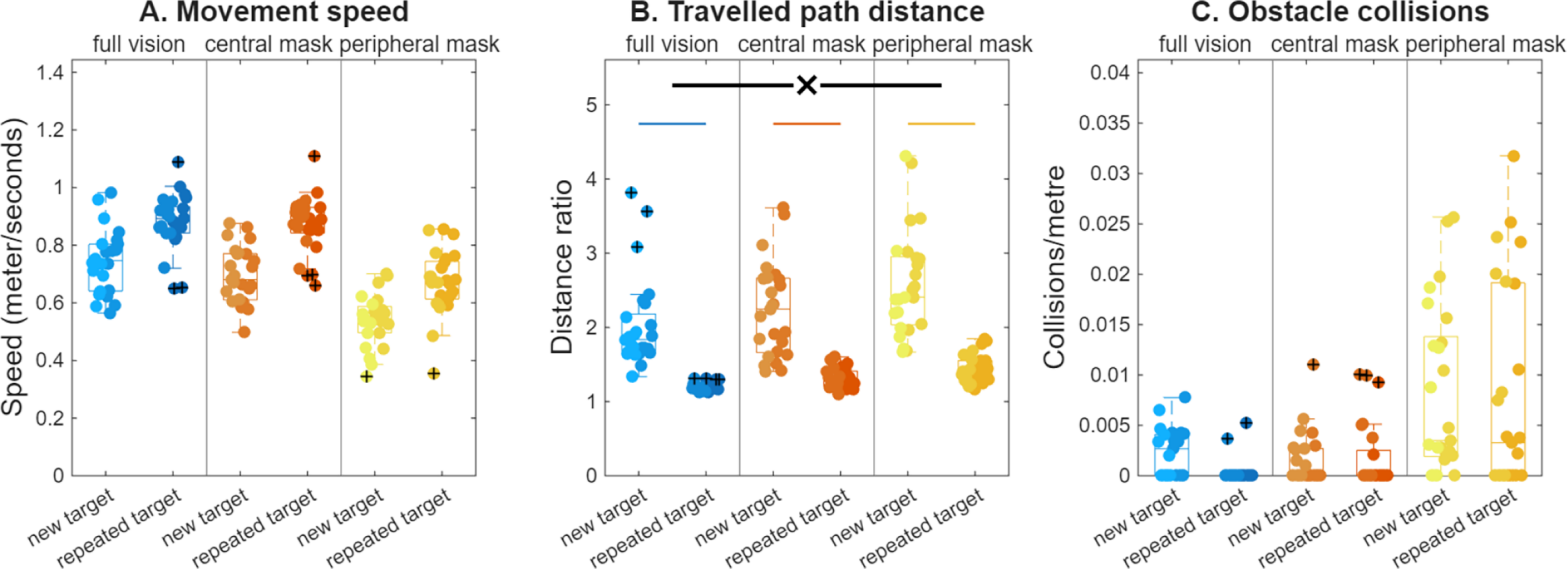
Navigation efficiency, with (**A**) movement speed, (**B**) travelled path distance normalized for the shortest path possible, and (**C**) the frequency of obstacle collisions, for new and repeated target searches across the three vision simulation conditions. For further details, see the caption to Figure 2.

Movement speed significantly increased for repeated targets, *F*(1, 65) = 298.41, *MSE* = 0.77, *P* < 0*.*001 (Fig. 3A), but did so similarly for the three vision conditions (full vision: Δ = 0.15 ± 0.08 m/s, central mask: Δ = 0.18 ± 0.06 m/s, peripheral mask: Δ = 0.13 ± 0.08 m/s), with the interaction not reaching significance, *F*(2, 65) = 2.56, *MSE* = 0.01, *P* = 0.09. The travelled path distance, normalized for the shortest path possible, decreased significantly overall for the repeated targets, *F*(1, 65) = 165.96, *MSE* = 27.08, *P* < 0*.*001 (Fig. 3B), although this interacted significantly with vision condition, *F*(2, 65) = 3.95, *MSE* = 0.64, *P* = 0.02. Path distance decreased significantly in each condition, full vision: Δ = −0.65 ± 0.25, *t*(17) = 11.07, *P* < 0*.*001; central mask: Δ = −0.92 ± 0.62, *t*(24) = 7.43, *P* < 0*.*001; peripheral mask: Δ = −1.14 ± 0.67, *t*(24) = 8.46, *P* < 0*.*001, although the decrease was significantly greater in the peripheral mask condition than for full vision, *F*(1, 41) = 8.83, *MSE* = 1.29, *P* = 0.01. The repetition-related reduction did not differ between the central mask and full vision conditions, *F*(1, 41) = 3.23, *MSE* = 0.41, *P* = 0.24, nor between the central and peripheral mask conditions, *F*(1, 48) = 1.40, *MSE* = 0.30, *P* = 0.73. Between the first and second halves, the actual average travelled distance per product search decreased by 8.5 ± 3.2 m with full vision, 10.5 ± 6.1 m with a central mask, and 12.4 ± 5.0 m with a peripheral mask. To give an illustration of the potential real-life implications—the average search path was 24.1 m in the first one-half of the experiment and 15.6 m in the second one-half of the experiment in the full vision condition. The average movement speed increased from 0.7 to 0.9 m/s. This increase in movement speed alone would have resulted in a 23% decrease in search time. The combination of the decrease in travelled distance and the increase in movement speed, resulted in a time saving of 50% for the second one-half of the experiment. The number of obstacle collisions showed no significant effect of target repetition, *F*(1, 66) = 2.16, *MSE* = 0.00, *P* = 0.15 (Fig. 3C), nor an interaction, *F*(2, 66) = 0.91, *MSE* = 0.00, *P* = 0.41. Thus, increased navigation efficiency likely contributed to the increased repetition benefits in overall task performance for the peripheral mask condition.

To conclude, learning-related improvements in task completion times and navigational efficiency were more prominent under conditions of vision loss and particularly for peripheral vision loss. We subsequently sought to trace the underlying source(s) of these learning benefits—that is, which components of behavior contributed to them. These analyses are therefore exploratory in nature, although they were explicitly planned as such (following Veerkamp et al.^9^). For these analyses, we present separately the data for the initiation phase (before starting the search), exploration phase (looking for the target), and homing-in phase (moving toward and grabbing the target once found).

### Initiation Phase

#### Duration

The initiation phase lasted from the start of the search until the first teleport was made. At the start of a sequence, participants heard and memorized the shopping list before starting the search for the first target product, thus naturally prolonging the initiation duration; therefore, we distinguished between the initiation phase for the first product search on the one hand, and for the second to fourth product searches on the other. The duration of the initiation phase in the first product search significantly decreased overall with repeated searches, *F*(1, 70) = 77.69, *MSE* = 3316.31, *P* < 0*.*001 (Fig. 1B), without further interaction with the vision condition, full vision: Δ = −9.4 ± 5.4 s, central mask: Δ = −9.5 ± 8.7 s, peripheral mask: Δ = −9.6 ± 12.4 s; *F*(2, 70) = 0.00, *MSE* = 0.10, *P* = 1.0. Similarly, the duration of the initiation phase in the second to fourth product searches was overall significantly reduced in repeated searches, *F*(1, 68) = 77.74, *MSE* = 81.27, *P* < 0*.*001 (Fig. 1C), again without further interaction with the vision condition, full vision: Δ = −1.7 ± 1.2 s, central mask: Δ = −1.0 ± 0.7 s, peripheral mask: Δ = −1.9 ± 2.0 s; *F*(2, 68) = 2.43, *MSE* = 2.54, *P* = 0.10. There was a significant interaction with frame rate however, *F*(2,65) = 4.42, *MSE* = 4.19, *P* = 0.02, reflecting that there was a stronger decrease in duration with a peripheral mask for participants with a higher frame rate (Supplementary Material B), suggesting that this group would benefit relatively more from repetition. However, given that this effect did not hold across all participants, we deem it safer to conclude that the search initiation stage did not substantially explain the vision by repetition interaction observed for the overall task performance.

#### Gaze and Other Orienting Behavior

**Figure 4.**
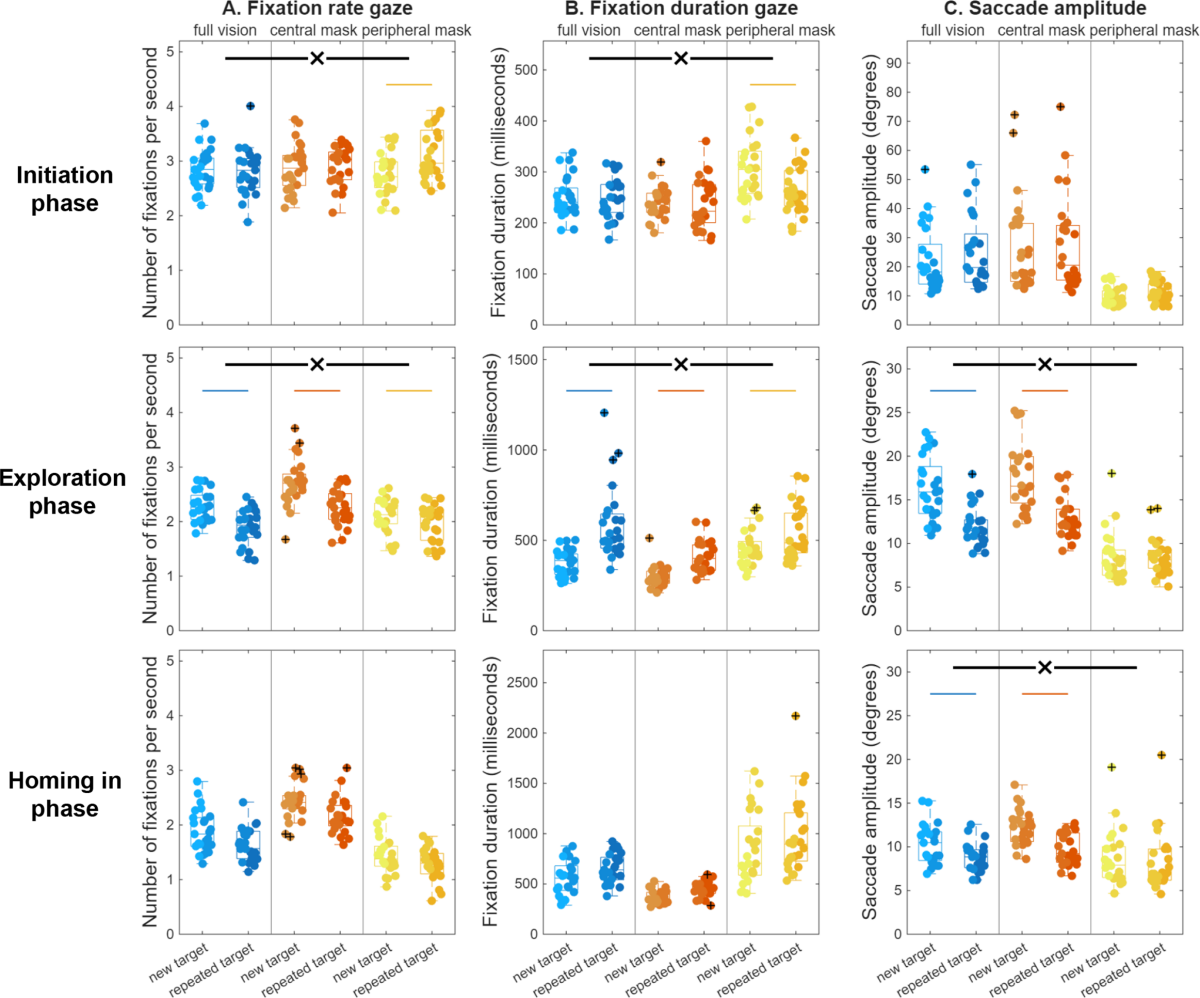
Gaze behavior in each of the three search phases, with (**A**) fixation rate, (**B**) fixation duration, and (**C**) saccade amplitude, for new and repeated target searches across the three vision simulation conditions. For further details, see the caption to Figure 2.

Fixation rate increased, and fixation duration decreased overall with repetition, *F*(1, 71) = 9.04, *MSE* = 0.45, *P* < 0*.*01; *F*(1, 71) = 10.99, *MSE* = 8024, *P* < 0*.*01 (Figs. 4A, 4B, respectively). For both, a significant interaction effect was observed, *F*(2, 71) = 11.89, *MSE* = 0.59, *P* < 0*.*001; *F*(2, 71) = 9.67, *MSE* = 7059, *P* < 0*.*001, respectively, and reflected the fact that these effects were present for the peripheral mask condition, but not the other vision conditions. Specifically, fixation rate increased significantly in the peripheral mask condition only, full vision: Δ = −0.05 ± 0.26 fixations/s, *t*(23.0) = 1.01, *P* = 0.97; central mask: Δ = 0.03 ± 0.37 fixations/s, *t*(24.0) = −0.36, *p =* 1.00; peripheral mask: Δ = 0.36 ± 0.30 fixations/s, *t*(24.0) = −6.00, *P* < 0*.*001, with greater increases compared with both full vision, *F*(1, 47) = 26.27, *MSE* = 1.04, *P* < 0*.*001, and the central mask condition, *F*(1, 48) = 12.10, *MSE* = 0.69, *P* < 0*.*01, with no significant difference between the full vision and central mask conditions, *F*(1, 47) = 0.76, *MSE* = 0.04, *P* = 1.0. There was a significant interaction effect of repetition, vision condition, and frame rate for fixation rate, *F*(2,68) = 4.55, *MSE* = 0.21, *P* = 0.01. The stronger increase in fixation rate for repeated targets in the peripheral mask condition was even stronger for the higher frame rate group, thus confirming the pattern (Supplementary Material B). Fixation duration only decreased for the peripheral mask condition, full vision: Δ = −0.90 ± 30.36 ms, *t*(24.0) = 0.15, *p =* 1.00; central mask: Δ = −1.03 ± 41.05 ms, *t*(23.0) = 0.12, *p =* 1.00; peripheral mask: Δ = −42.26 ± 42.24 ms, *t*(24.0) = 5.00, *P* < 0*.*001. The fixation duration decreased significantly more in the peripheral mask condition compared with the full vision, *F*(1, 48) = 15.81, *MSE* = 10694, *P* < 0*.*001, and central mask conditions, *F*(1, 47) = 11.99, *MSE* = 10408, *P* < 0*.*01, although there was no significant difference between the full vision and central mask conditions, *F*(1, 47) = 0.00, *MSE* = 0.11, *P* = 1.0. Here, repetition, vision condition, and frame rate also showed a significant interaction effect, *F*(2,68) = 3.26, *MSE* = 2263, *P* = 0.04. Again, the decrease in fixation duration for repeated targets in the peripheral mask condition was stronger for the higher frame rate group, thus confirming the pattern (Supplementary Material B). Saccade amplitudes were overall significantly larger for repeated targets, *F*(1, 70) = 8.03, *MSE* = 182.54, *P* < 0*.*01 (Fig. 4C), without this being further modulated by an interaction effect with vision condition, full vision: Δ = 2.9 ± 6.9 deg, central mask: Δ = 2.6 ± 9.1 deg, peripheral mask: Δ = 1.2 ± 2.8 deg; *F*(2, 70) = 0.47, *MSE* = 10.69, *P* = 0.63.

**Figure 5.**
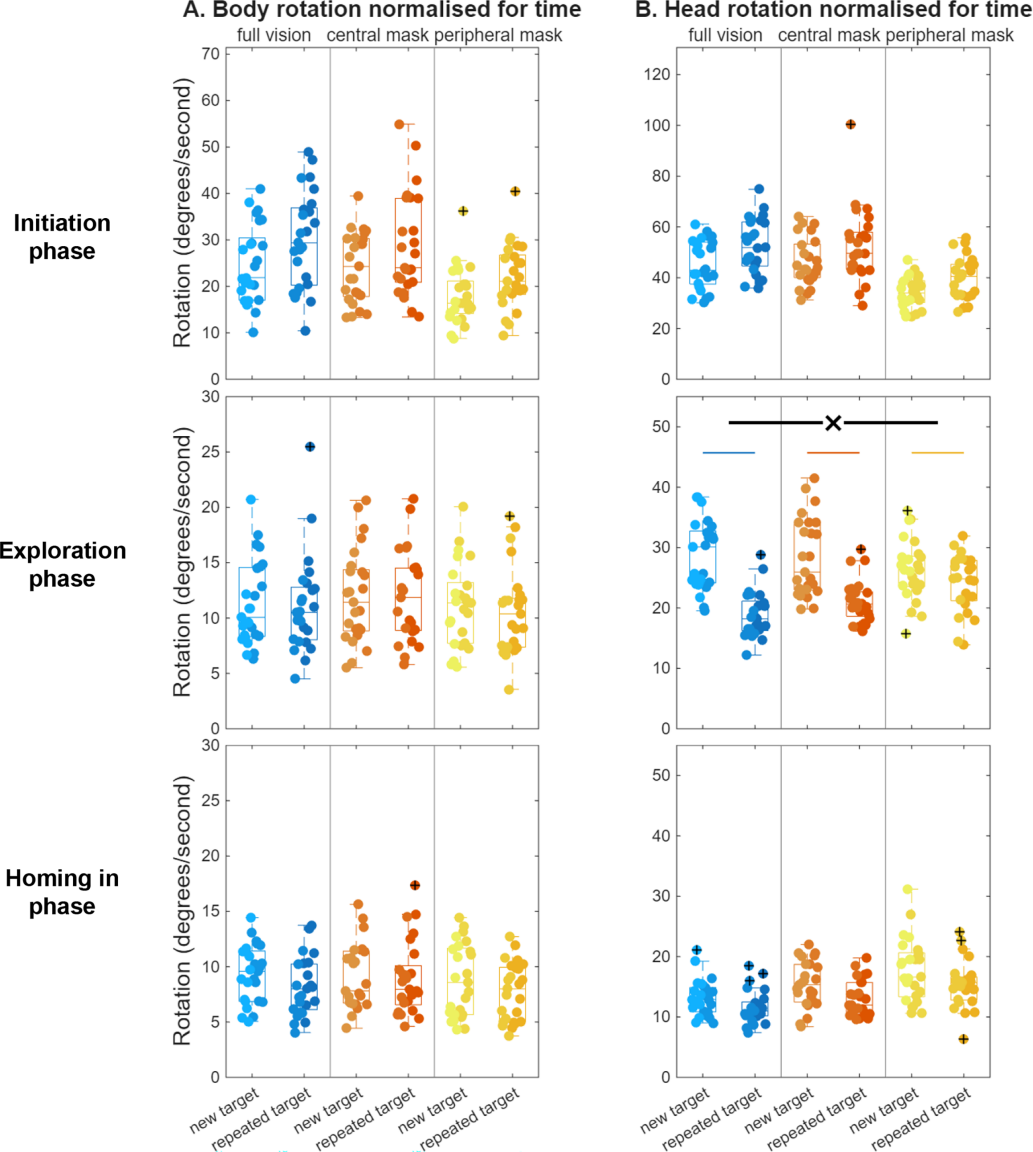
Other orienting behavior in each of the three search phases, with (**A**) body rotation, and (**B**) head rotation, for new and repeated target searches across the three vision simulation conditions. For further details, see the caption to Figure 2.

During the initiation phase, both the body and the head rotated more for repeated targets, *F*(1, 72) = 54.53, *MSE* = 809.75, *P* < 0*.*001; *F*(1, 71) = 56.52, *MSE* = 1474.28, *P* < 0*.*001 (Figs. 5A, 5B, respectively), with no significant interaction with vision condition, body: full vision: Δ = 5.3 ± 5.0 deg/s, central mask: Δ = 4.6 ± 7.4 deg/s, peripheral mask: Δ = 4.1 ± 3.2 deg/s; *F*(2, 72) = 0.29, *MSE* = 4.27, *P* = 0.75; head: full vision: Δ = 7.6 ± 7.6 deg/s, central mask: Δ = 5.4 ± 7.6 deg/s, peripheral mask: Δ = 5.9 ± 6.5 deg/s; *F*(2, 71) = 0.58, *MSE* = 15.13, *P* = 0.56, respectively.

Thus, repetition came with increased gaze-based orienting-related rotations during the initiation phase when peripheral vision was masked.

### Exploration Phase

#### Duration

The exploration phase spanned the period from the first teleport up to the first fixation on the target product. The duration of the exploration phase reduced substantially for repeated targets compared with new targets, *F*(1, 66) = 456.53, *MSE* = 22104.20, *P* < 0*.*001 (Fig. 2D). The interaction effect with vision condition was significant, *F*(2, 66) = 10.98, *MSE* = 531.58, *P* < 0*.*001. The exploration phase duration decreased with repetition in each of the vision conditions, full vision: Δ = −19.2 ± 7.5 s, *t*(21) = 12.01, *P* < 0*.*001; central mask: Δ = −24.0 ± 10.0 s, *t*(24) = 12.03, *P* < 0*.*001; peripheral mask: Δ = −32.9 ± 11.6 s, *t*(21) = 13.30, *P* < 0*.*001, but this decrease was significantly greater in the peripheral mask condition than in the full vision condition, *F*(1, 42) = 21.67, *MSE* = 1031.47, *P* < 0*.*001, and the central mask condition, *F*(1, 45) = 7.89, *MSE* = 457.36, *P* = 0.02. The repetition-related decrease in the exploration phase duration did not differ significantly between the full vision and the central mask conditions, *F*(1, 46) = 3.47, *MSE* = 137.82, *P* = 0.21.

### Gaze and Other Orienting Behavior

The fixation rate decreased significantly for repeated targets, *F*(1, 69) = 85.22, *MSE* = 3.55, *P* < 0*.*001 (Fig. 4A), with a significant interaction effect with vision condition, *F*(2, 69) = 7.69, *MSE* = 0.32, *P* < 0*.*001. The fixation rate decreased in each of the vision conditions, full vision: Δ = −0.38 ± 0.28 fixations/s, *t*(24) = 6.93, *P* < 0*.*001; central mask: Δ = −0.43 ± 0.34 fixations/s, *t*(21) = 5.96, *P* < 0*.*001; peripheral mask: Δ = −0.13 ± 0.25 fixations/s, *t*(24) = 2.59, *P* = 0.05, but less so in the peripheral mask condition than in the full vision and central mask conditions, *F*(1, 48) = 11.63, *MSE* = 0.40, *P* < 0*.*01, *F*(1, 45) = 12.39, *MSE* = 0.54, *P* < 0*.*01, respectively, with no difference between the latter two, *F*(1, 45) = 0.31, *MSE* = 0.01, *P* = 1.0. There was a significant further interaction with the frame rate, *F*(2,66) = 3.76, *MSE* = 0.13, *P* = 0.03, because the decrease in fixation rate with repetition for the central mask condition was more pronounced for the higher frame rate group, again confirming the pattern (Supplementary Material B). Also, fixation durations were significantly longer for repeated targets, *F*(1, 66) = 118.29, *MSE* = 510454, *P* < 0*.*001 (Fig. 4B), and also here the interaction effect with vision condition was significant, *F*(2, 66) = 3.37, *MSE* = 14562, *P* = 0.04. Fixation duration increased with repetition in each of the vision conditions, full vision: Δ = 161.87 ± 87.87 ms, *t*(21) = −8.64, *P* < 0*.*001; central mask: Δ = 111.15 ± 63.21 ms, *t*(23) = −8.61, *P* < 0*.*001; peripheral mask: Δ = 92.12 ± 119.76 ms, *t*(20) = −3.69, *P* < 0*.*01, with trends toward smaller increases in the central mask and the peripheral mask conditions compared with full vision, *F*(1, 44) = 5.11, *MSE* = 14760, *P* = 0.09, *F*(1, 43) = 4.92, *MSE* = 27348, *P* = 0.10, respectively. The increase in fixation duration with repetition did not differ between the central mask and peripheral mask conditions, *F*(1, 45) = 0.47, *MSE* = 2127, *P* = 1.00. Saccades were smaller in repeated searches, *F*(1, 70) = 105.31, *MSE* = 365.67, *P* < 0*.*001 (Fig. 4C), and this too interacted with vision condition, *F*(2, 70) = 25.51, *MSE* = 88.59, *P* < 0*.*001. Saccades became smaller with repetition only in the full vision and central mask conditions, and not with a peripheral mask, full vision: Δ = −4.62 ± 2.73 deg, *t*(23) = 8.28, *P* < 0*.*001; central mask: Δ = −4.83 ± 3.22 deg, *t*(24) = 7.50, *P* < 0*.*001; peripheral mask: Δ = −0.04 ± 1.68 deg, *t*(23) = 0.12, *P* = 1.00. Indeed, the decrease in saccade amplitude with repetition was significantly smaller in the peripheral mask condition than in the full vision condition, *F*(1, 46) = 48.87, *MSE* = 125.83, *P* < 0*.*001, and than in the central mask condition, *F*(1, 47) = 42.01, *MSE* = 140.43, *P* < 0*.*001, although this decrease did not differ between the full vision and the central mask conditions, *F*(1, 47) = 0.06, *MSE* = 0.27, *P* = 1.00.

Body rotation did not show a significant effect of repetition, *F*(1, 70) = 2.52, *MSE* = 6.87, *P* = 0.12 (Fig. 5A). However, the head rotated less in repeated searches, *F*(1, 68) = 104.45, *MSE* = 1514.03, *P* < 0*.*001 (Fig. 5B), with a significant interaction effect with vision condition, *F*(2, 68) = 14.25, *MSE* = 206.63, *P* < 0*.*001. Head rotation decreased with repetition in each of the vision conditions, full vision: Δ = −10.2 ± 5.7 deg/s, *t*(230) = 8.70, *P* < 0*.*001; central mask: Δ = −7.5 ± 6.7 deg/s, *t*(23) = 5.46, *P* < 0*.*001; peripheral mask: Δ = −1.9 ± 2.9 deg/s, *t*(22) = 3.25, *P* = 0.01. This repetition-related decrease in head rotations was significantly less with a peripheral mask than with full vision, *F*(1, 45) = 38.47, *MSE* = 398.71, *P* < 0*.*001, and than with a central mask, *F*(1, 45) = 13.43, *MSE* = 181.57, *P* < 0*.*01, and did not differ between full vision and a central mask, *F*(1, 46) = 2.21, *MSE* = 43.07, *P* = 0.43.

Thus, in contrast with the initiation phase, where most changes in orienting behavior occurred for the peripheral mask condition, during the exploration phase the central mask and control full vision conditions actually changed most. Under those conditions, orienting behavior became considerably more efficient with repetition, as reflected by fewer orienting movements. However, note that in the peripheral mask condition there were already fewer orienting movements to begin with. We return to this in the Discussion.

### Homing-in Phase

#### Duration

The homing-in phase was the time period between the first fixation on the target product and actually selecting that product with the controller. The duration of the homing-in phase significantly decreased from new searches to repeated searches, *F*(1, 66) = 56.22, *MSE* = 507.74, *P* < 0*.*001, (Fig. 2E), without further interaction with vision condition (full vision: Δ = −2.3 ± 2.1 s, central mask: Δ = −4.0 ± 3.7 s, peripheral mask: Δ = −5.2 ± 6.0 s), although there was a trend toward larger repetition benefits in the peripheral mask condition, *F*(2, 66) = 2.77, *MSE* = 25.02, *P* = 0.07. There was an interaction between repetition, vision condition, and frame rate however, *F*(2,63) = 3.93, *MSE* = 31.64, *P* = 0.02, reflecting the fact that in the central mask condition, repetition benefits were somewhat stronger in the high frame rate group (Supplementary Material B). Because this did not occur in the overall group, we do not wish to draw strong conclusions from this interaction.

#### Gaze and Other Orienting Behavior

There were fewer fixations but with longer duration for the repeated searches, *F*(1, 68) = 61.05, *MSE* = 1.93, *P* < 0*.*001; *F*(1, 69) = 22.05, *MSE* = 290776, *P* < 0*.*001 (Figs. 4A, 4B, respectively), without a significant interaction with vision condition, rate: full vision: Δ = −0.25 ± 0.23 fixations/s, central mask: Δ = −0.30 ± 0.33 fixations/s, peripheral mask: Δ = −0.15 ± 0.19 fixations/s, *F*(2, 68) = 2.12, *MSE* = 0.07, *P* = 0.13; duration: full vision: Δ = 97 ± 100 ms, central mask: Δ = 71 ± 61 ms, peripheral mask: Δ = 101 ± 255 ms, *F*(2, 69) = 0.24, *MSE* = 3136, *P* = 0.79, respectively. Saccades were smaller for repeated searches, *F*(1, 70) = 41.85, *MSE* = 78.53, *P* < 0*.*001 (Fig. 4C), and this interacted with vision condition, *F*(2, 70) = 6.92, *MSE* = 12.98, *P* < 0*.*01. Saccade amplitudes became smaller with repetition under full vision and a central mask, but not under a peripheral mask, full vision: Δ = −1.5 ± 1.8 s, *t*(24) = 4.28, *P* < 0*.*001; central mask: Δ = −2.5 ± 1.7 s, *t*(24) = 7.27, *P* < 0*.*001; peripheral mask: Δ = −0.4 ± 2.3 s, *t*(22) = 0.83, *P* = 1.00. The decrease in saccade amplitudes was significantly greater with a central mask than with a peripheral mask, *F*(1, 46) = 12.77, *MSE* = 25.95, *P* < 0*.*01, but this decrease did not differ significantly between full vision and the central mask condition, *F*(1, 48) = 3.82, *MSE* = 5.80, *P* = 0.17, or between full vision and the peripheral mask condition, *F*(1, 46) = 3.57, *MSE* = 7.49, *P* = 0.20.

Body and head rotations both significantly decreased for repeated targets, *F*(1, 71) = 11.84, *MSE* = 24.88, *P* < 0*.*001; *F*(1, 67) = 30.99, *MSE* = 156.03, *P* < 0*.*001 (Figs. 5A, 5B, respectively), without a significant interaction with vision condition, body: full vision: Δ = −1.16 ± 2.35 deg/s, central mask: Δ = −0.29 ± 1.87 deg/s, peripheral mask: Δ = −1.01 ± 1.89 deg/s, *F*(2, 71) = 1.27, *MSE* = 2.66, *P* = 0.29; head: full vision: Δ = −2.0 ± 2.2 deg/s, central mask: Δ = −2.3 ± 3.5 deg/s, peripheral mask: Δ = −2.1 ± 3.6 deg/s, *F*(2, 67) = 0.09, *MSE* = 0.43, *P* = 0.92.

Thus, with repetition, gaze-related behavior became more efficient (i.e., fewer/smaller movements) especially in the central mask and control conditions. However, here too in the peripheral mask condition there were already fewer orienting movements to begin with.

### New Targets in a Familiar Environment

So far we have assessed learning by comparing repeated with first-time searches. Changes may then reflect learning the target location, and/or learning the environmental lay-out of the supermarket itself. To assess the contribution of the latter, we compared sequence 2, in which participants searched for four new products but within the same environment, with sequence 1 (searching for four different products). Directly comparing these sequences allowed us to assess whether the participants had learned something about the environment's context during the first sequence that would benefit them during the second sequence. However, we found no sign of improved task performance from sequence 1 to sequence 2. Task completion times and the movement speed hardly changed, completion time: *F*(1,69) = 1.05, *MSE* = 201.43, *P* = 0.31; movement speed: *F*(1,71) = 3.43, *MSE* = 0.01, *P* = 0.68, and if anything the travelled path distance increased for the second sequence, *F*(1,65) = 20.78, *MSE* = 9.89, *P* < 0*.*001, which was likely due to sequence 2 containing a search with a relatively short optimal path, resulting in small deviations being penalized relatively heavily. Given the lack of any effects and interactions with vision condition, we did not further interpret underlying changes in gaze and orienting behavior.

The lack of improvement from sequence 1 to sequence 2 also argues against the improvements that we observed between sequences 1 and 2 and sequences 3 and 4 being the result of generic factors unrelated to spatial learning: the VR apparatus, becoming quicker at pressing the button, and so on.

## Discussion

We assessed the degree to which spatial learning mitigates the consequences of simulated central and peripheral vision loss in a naturalistic visual search task. Performance improved substantially in all vision conditions when looking for previously searched-for targets, with decreases in the order of several tens of seconds in absolute terms and 50% in relative terms compared with when searching for new targets. Importantly, improvements in task completion times and navigational efficiency were more prominent under conditions of vision loss, and particularly peripheral vision loss, highlighting the contribution of spatial learning in mitigating the consequences of decreased vision.

Our findings confirm and extend those of previous studies that looked at learning with normal vision. In line with earlier studies,^1^^–^^4^ we observed shorter search times and shorter paths for repeated searches in the absence of vision impairment. We also observed fewer and longer fixations, as well as increased movement speeds (as was also reported by Aivar et al.).^4^ Furthermore, in agreement with Li et al.,^2^ for most of the search process head rotations decreased for repeated searches, even after normalizing for the shorter search times. However, here we also showed that, during the initiation phase of the search, orienting-related rotations (i.e., body and head gaze rotations) generally increased with repetition. This finding likely reflects people first orienting themselves toward the now known direction in which they should head before they leave the starting point, rather than just starting to move, as would be expected if the target direction was not yet known. In contrast, in the exploration and homing-in phases, which spanned the periods from the first movement (i.e., teleport) up to actually selecting the target, orienting-related rotations generally decreased in the repeated searches. This result is consistent with participants knowing where to go, the search thus being more targeted, and requiring less scanning of the environment.

The improvements in performance for repeated searches were the result of learning where in space the specific target was located, and not necessarily the result of learning a contextual layout of the specific supermarket environment or of familiarization with the test equipment we used. There was a lack of improvement for new targets in a familiar environment (between the first two search sequences), which is consistent with findings from Li et al.,^2^ and suggests little incidental learning of the environment itself. However, this finding differs from studies by Aivar et al.^4^ and Beitner et al.,^13^ who did observe incidental learning. In those studies, the targets were simple geometrical shapes that were in principle unrelated to the scene context, and so they may have stood out more both in terms of perceptual saliency and memory once incidentally encountered. In our study, the target products were intrinsically part of the environment, and may have thus garnered little attention when not the actual target. Moreover, in Beitner et al.,^13^ the VR environment was an apartment much smaller than our supermarket (3.5 × 3.8 m vs 25.0 × 35.0 m), which may also have caused more effective learning. What Beitner et al.^13^ further showed was that, if such generic learning occurs, it does so early, in their case from the first to the second trial. Thus, the lack of such effects in our study goes against the idea that the learning effects we observed for the repeated searches in the second one-half of the experiment simply reflected overall performance improvements not caused by spatial learning. Moreover, travelled path efficiencies clearly improved with repeated searches, and this measure is not simply explained by generic aspects such as quicker button presses. Note here that it is the target locations that were learned, and not the routes; although the targets repeated, the starting points, and therefore the routes toward the targets, did not.

Importantly, performance improved more for repeated searches under conditions of vision loss than under full vision conditions, with greater decreases in task completion times and travelled paths. These improvements were most prominent for the peripheral vision loss condition, and to a lesser extent for central vision loss. This result likely reflects the fact that there was a greater capacity for improvement with vision loss, and particularly with peripheral vision loss, given that participants initially performed worse in those conditions than in the full vision condition. However, and importantly, this finding also implies that performance for repeated searches with vision loss is far better than for new searches. In fact, performance in the repeated search approached that with full vision. The substantial decrease in travelled distances, combined with the increase in movement speed, resulted in a substantial decrease in the time needed to conduct a search. In real life, this would translate to valuable savings in time and energy, which is especially relevant given that people with vision impairment generally experience more fatigue.^14^ This finding highlights the particularly important role of spatial learning for people with a vision impairment, who cannot make as much use of real-time visual information, and instead must rely relatively more on memory. Overall, this finding emphasizes the need for consistently structured environments for people with impaired vision.

With central vision loss, the effects of spatial learning on orienting behavior were overall very similar as for full vision, showing more orienting-related rotations in the initiation phase and fewer rotations in the exploration and homing-in phases for repeated searches. The rotations of the eyes and gaze in the exploration and homing-in phases decreased slightly more for repeated searches with central vision loss than with full vision. Initially, eye and gaze rotations with central vision loss were greater for new searches, so the stronger decreases in rotation might again reflect a greater capacity for change.

Learning-related orienting behavior showed a different profile in the peripheral vision loss condition. Here eye rotations increased more in the initiation phase, with more and shorter fixations, possibly because peripheral vision is not available to guide the participant into which direction they should begin to move, and thus more scanning is required with the remaining central vision. Furthermore, in the exploration and homing-in phases, rotations did not decrease as much for repeated searches as in the other conditions. This may reflect a floor effect, because saccades were already smaller with peripheral vision loss to begin with, and a further decrease in saccadic amplitude may not be functional. Likely, some minimum amount of scanning is required to still move through an environment, even when the environment is familiar. The finding that scanning did become more efficient with repetition in the central vision loss condition, but not in the peripheral vision loss condition, although in both conditions the movement speed increased similarly, underlines the likely importance of peripheral vision for moving through an environment. This finding aligns with the finding that the reductions in head rotations were also not as pronounced with peripheral vision loss, even though there was no floor effect there, because the remaining head movements would be required to compensate for the loss of peripheral vision that is usually used for navigating an environment. Interestingly, people with retinitis pigmentosa, a condition resulting in peripheral vision loss, have been reported to make saccades of a similar amplitude as normal-sighted controls, resulting in saccades directed toward positions that were outside of their residual visual field, which may reflect a strategy for dealing with the peripheral vision loss.^15^^,^^16^ Participants in our experiment with simulated peripheral vision loss did initially make saccades mostly within their remaining visual field, but may have partly learned to adopt the same strategy to increase the saccade amplitudes, which would go against any further reduction in saccadic amplitude with repetition. Finally, the fact that orienting did not become as efficient as for the other conditions could also mean that spatial learning was less precise with simulated peripheral vision loss. This result would be in line with previous studies showing that spatial learning is impaired with peripheral vision loss. Furthermore, people with restricted peripheral vision have been shown to make larger errors in pointing out the location of earlier encountered landmarks.^5^^,^^6^ However, we did observe a strong improvement in performance for repeated searches, even with peripheral vision being masked. It is worth noting that both can be true at the same time: vision loss may impede learning, but what is learned could be of much more help. Moreover, our supermarket environment provided more contextual information that was likely to be more familiar to participants than the more ad hoc museum and hallway environments used in previous studies, and this context may have aided spatial learning. Hence, even though observers under peripheral mask conditions never quite reached the performance levels achieved with full vision, it is clear that they could obtain meaningful spatial information through learning.

In this study, we simulated visual field loss in people without impairment, and it remains reasonable to ask what the results tell us when compared with a comparable study testing participants with actual vision impairment. First, in our study we are able to examine the impact of the raw impairment without the influence of any longer-term adaptation to the impairment. People with vision impairment can be very good at adapting to their impairment,^17^^,^^18^ and the nature of the adaptation can be quite idiosyncratic between individuals. Here we can control for that long-term adaptation. Our results are particularly pertinent when considering the impact of newly acquired vision loss. The information learned can be very informative, for instance, for rehabilitation specialists who might want to emphasize spatial learning when helping individuals with newly acquired visual field loss to adapt to their impairment. Second, our approach allows us to control the nature of the vision loss. As clinicians know, no two forms of partial visual field loss are ever the same. Therefore, a study of people with actual impairment will naturally have considerable variability in the nature of the vision loss between individuals. In our study, we are better able to isolate the impact of specific types of visual field loss. Nonetheless, it does remain important to understand the impact of spatial learning on individuals with actual impairment. Fortunately, at the time of writing, we are in the process of testing a group of individuals with actual vision loss on the same task. We hope to be able to report the findings of that study in the not too distant future.

Our results do emphasize the need for designing public spaces in such a way that spatial memory can be used consistently to aid people with vision impairment. For example, persons with vision impairment have indicated that they often rely on cities having predictable layouts while navigating.^19^ Furthermore, people with vision impairment often receive orientation and mobility training that encourages the use of landmarks.^20^ However, consistency can be limited, because spaces are often managed by varying designers with different styles. Even within the same environment, consistency is not guaranteed. For example, supermarkets may change product locations and even general layouts for commercial reasons. This lack of consistency has been reported to be disorientating for both interior and exterior environments,^19^^,^^21^ and a clear recommendation is to incorporate the needs of the visually impaired into decision-making processes, for example, through active participatory schemes.^22^^,^^23^

We have shown that vision loss results in inefficient search in a naturalistic 3D environment, but that the consequences are largely mitigated by spatial learning. This is especially the case for peripheral vision loss, where both the costs of the impaired vision, and the learning benefits were greatest. Moreover, learning comes with vision loss-specific changes in orienting strategies in different stages during the search, as reflected in eye, head and body movements. The findings emphasize the adaptive nature of vision, the vital role of spatial learning, and the beneficial effects of consistent environments for people with vision loss.

## Supplementary Material

Supplement 1

Supplement 2

Supplement 3

Supplement 4

Supplement 5
