# Supplementary material for: Spatial Learning in Naturalistic Search With Simulated Vision Loss
Source: Transl Vis Sci Technol. 2026 Apr 14;15(4):10. doi: 10.1167/tvst.15.4.10 (PMC13089662; doi:10.1167/tvst.15.4.10)
Supplement: Supplement 1 [file tvst-15-4-10_s001.docx]

# Supplementary material A

Table An overview of the results for all outcome variables for new and repeated target searches, averaged across participants per vision condition. Outliers were excluded from the reported means and standard deviations (see Method). Post-hoc tests were only performed when the interaction effect was significant.

| category | variable |  | mean ± standard deviation | | | | | | main effect | interaction effect | post hoc tests | | |  |  |  |
| --- | --- | --- | --- | --- | --- | --- | --- | --- | --- | --- | --- | --- | --- | --- | --- | --- |
|  | name | unit | full vision | | central mask | | peripheral mask | | target repetition | target repetition x vision condition | new vs repeated target | | | Interaction effect target repetition x vision condition | | |
|  |  |  | new targets | repeated targets | new targets | repeated targets | new targets | repeated targets |  |  | full vision | central mask | peripheral mask | full vision vs central mask | full vision vs peripheral mask | central mask vs peripheral mask |
| task completion time | task completion time | seconds | 36.2 ± 6.8 | 16.8 ± 1.1 | 42.7 ± 10.0 | 19.3 ± 3.1 | 63.3 ± 10.7 | 27.7 ± 5.6 | <0.001 | <0.001 | <0.001 | <0.001 | <0.001 | 0.36 | <0.001 | <0.001 |
| navigation | movement speed | meters per second | 0.7 ± 0.1 | 0.9 ± 0.1 | 0.7 ± 0.1 | 0.9 ± 0.1 | 0.5 ± 0.1 | 0.7 ± 0.1 | <0.001 | 0.09 | - | - | - | - | - | - |
|  | travelled path distance | distance ratio | 1.8 ± 0.3 | 1.2 ± 0.0 | 2.2 ± 0.6 | 1.3 ± 0.1 | 2.6 ± 0.7 | 1.5 ± 0.2 | <0.001 | 0.02 | <0.001 | <0.001 | <0.001 | 0.24 | 0.01 | 0.73 |
|  | obstacle collisions | number of collisions divided by distance | 0.0 ± 0.0 | 0.0 ± 0.0 | 0.0 ± 0.0 | 0.0 ± 0.0 | 0.0 ± 0.0 | 0.0 ± 0.0 | 0.15 | 0.41 | - | - | - | - | - | - |
| initiation | duration first search | seconds | 29.8 ± 6.6 | 20.3 ± 5.6 | 32.9 ± 11.3 | 23.4 ± 7.5 | 38.8 ± 19.8 | 29.2 ± 11.5 | <0.001 | 1.00 | - | - | - | - | - | - |
|  | duration next searches | seconds | 4.2 ± 1.6 | 2.5 ± 0.8 | 3.5 ± 0.8 | 2.5 ± 0.8 | 7.1 ± 2.1 | 5.2 ± 2.0 | <0.001 | 0.10 | - | - | - | - | - | - |
|  | fixation rate | fixations per second | 2.8 ± 0.3 | 2.8 ± 0.3 | 2.9 ± 0.4 | 2.9 ± 0.3 | 2.8 ± 0.4 | 3.1 ± 0.5 | <0.01 | <0.001 | 0.97 | 1.00 | <0.001 | 1.00 | <0.001 | <0.01 |
|  | fixation duration | milliseconds | 249.8 ± 40.5 | 248.9 ± 40.3 | 236.0 ± 27.1 | 235.0 ± 48.5 | 306.0 ± 58.8 | 263.7 ± 45.6 | <0.01 | <0.001 | 1.00 | 1.00 | <0.001 | 1.00 | <0.001 | <0.01 |
|  | saccade amplitude | degrees | 20.9 ± 9.2 | 23.8 ± 10.9 | 25.0 ± 14.0 | 27.6 ± 16.6 | 9.8 ± 3.1 | 11.0 ± 3.6 | <0.01 | 0.63 | - | - | - | - | - | - |
|  | body rotation | degrees per second | 24.7 ± 8.4 | 30.0 ± 10.4 | 24.1 ± 7.5 | 28.6 ± 11.0 | 18.0 ± 6.0 | 22.1 ± 7.0 | <0.001 | 0.75 | - | - | - | - | - | - |
|  | head rotation | degrees per second | 45.1 ± 9.5 | 52.7 ± 10.7 | 45.3 ± 8.8 | 50.8 ± 10.6 | 34.1 ± 6.1 | 40.1 ± 8.3 | <0.001 | 0.56 | - | - | - | - | - | - |
| exploration | duration | seconds | 26.7 ± 7.6 | 7.5 ± 1.2 | 33.7 ± 11.1 | 9.6 ± 2.7 | 46.7 ± 11.8 | 13.8 ± 4.2 | <0.001 | <0.001 | <0.001 | <0.001 | <0.001 | 0.21 | <0.001 | 0.02 |
|  | fixation rate | fixations per second | 2.3 ± 0.3 | 1.9 ± 0.3 | 2.7 ± 0.3 | 2.3 ± 0.3 | 2.1 ± 0.3 | 2.0 ± 0.3 | <0.001 | <0.001 | <0.001 | <0.001 | 0.05 | 1.00 | <0.01 | <0.01 |
|  | fixation duration | milliseconds | 367.0 ± 73.9 | 528.9 ± 109.7 | 288.6 ± 41.1 | 399.8 ± 77.9 | 436.3 ± 76.9 | 528.4 ± 146.8 | <0.001 | 0.04 | <0.001 | <0.001 | <0.01 | 0.09 | 0.10 | 1.00 |
|  | saccade amplitude | degrees | 16.3 ± 3.7 | 11.7 ± 1.9 | 17.7 ± 4.0 | 12.9 ± 2.4 | 8.2 ± 2.8 | 8.2 ± 1.9 | <0.001 | <0.001 | <0.001 | <0.001 | 1.00 | 1.00 | <0.001 | <0.001 |
|  | body rotation | degrees per second | 11.0 ± 3.7 | 10.5 ± 3.2 | 11.9 ± 4.2 | 11.9 ± 4.1 | 11.0 ± 3.7 | 10.2 ± 3.5 | 0.12 | 0.53 | - | - | - | - | - | - |
|  | head rotation | degrees per second | 28.6 ± 5.8 | 18.4 ± 3.2 | 28.1 ± 6.5 | 20.7 ± 3.1 | 26.3 ± 4.3 | 24.3 ± 4.5 | <0.001 | <0.001 | <0.001 | <0.001 | 0.01 | 0.43 | <0.001 | <0.01 |
| homing in | duration | seconds | 9.1 ± 2.1 | 6.8 ± 0.8 | 11.4 ± 3.6 | 7.4 ± 1.6 | 15.8 ± 4.6 | 10.6 ± 3.5 | <0.001 | 0.07 | - | - | - | - | - | - |
|  | fixation rate | fixations per second | 1.9 ± 0.4 | 1.6 ± 0.3 | 2.4 ± 0.2 | 2.1 ± 0.3 | 1.4 ± 0.3 | 1.3 ± 0.3 | <0.001 | 0.13 | - | - | - | - | - | - |
|  | fixation duration | milliseconds | 556.7 ± 169.6 | 653.8 ± 145.4 | 371.8 ± 66.3 | 443.0 ± 63.4 | 826.4 ± 349.1 | 927.9 ± 290.1 | <0.001 | 0.79 | - | - | - | - | - | - |
|  | saccade amplitude | degrees | 10.3 ± 2.3 | 8.8 ± 1.6 | 12.2 ± 2.0 | 9.7 ± 1.8 | 8.1 ± 2.0 | 7.7 ± 2.1 | <0.001 | <0.01 | <0.001 | <0.001 | 1.00 | 0.17 | 0.20 | <0.01 |
|  | body rotation | degrees per second | 9.3 ± 2.6 | 8.1 ± 2.6 | 8.9 ± 2.8 | 8.6 ± 2.8 | 8.8 ± 3.3 | 7.7 ± 2.6 | <0.001 | 0.29 | - | - | - | - | - | - |
|  | head rotation | degrees per second | 13.2 ± 3.1 | 11.3 ± 2.4 | 15.4 ± 3.8 | 13.1 ± 3.0 | 17.2 ± 4.3 | 15.1 ± 2.7 | <0.001 | 0.92 | - | - | - | - | - | - |
