# Supplementary material for: Spatial Learning in Naturalistic Search With Simulated Vision Loss
Source: Transl Vis Sci Technol. 2026 Apr 14;15(4):10. doi: 10.1167/tvst.15.4.10 (PMC13089662; doi:10.1167/tvst.15.4.10)

# Supplementary material B

## Initiation phase duration

### Lower frame rate Higher frame rate


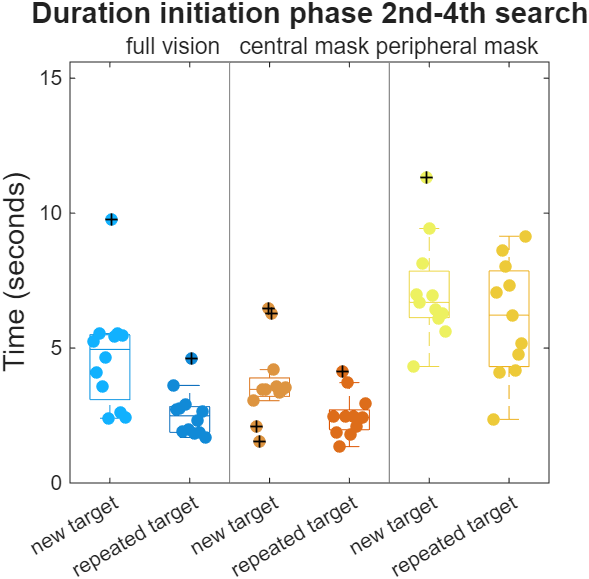

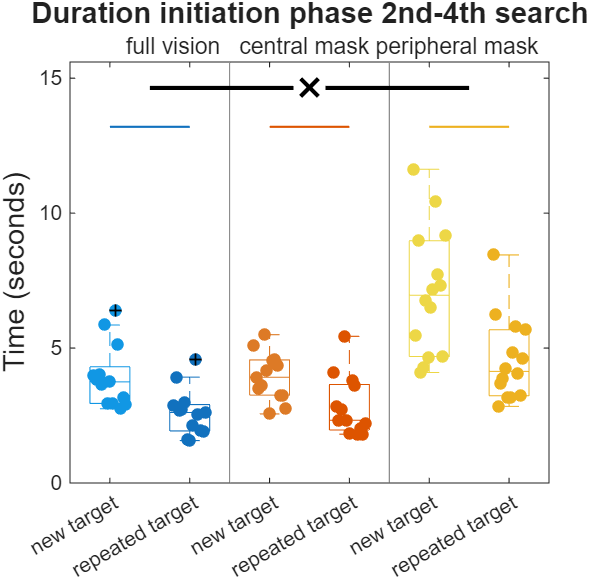


## Initiation phase fixation rate

### Lower frame rate Higher frame rate


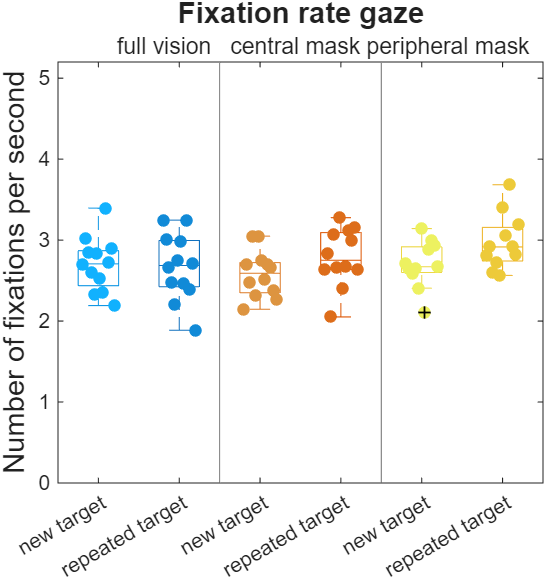

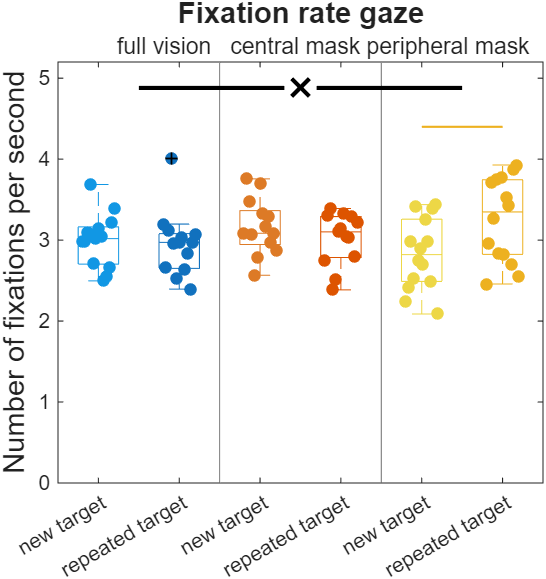


## Initiation phase fixation duration

### Lower frame rate Higher frame rate


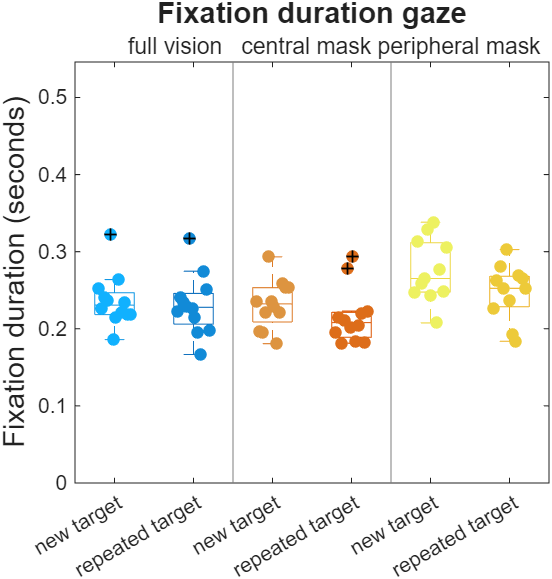

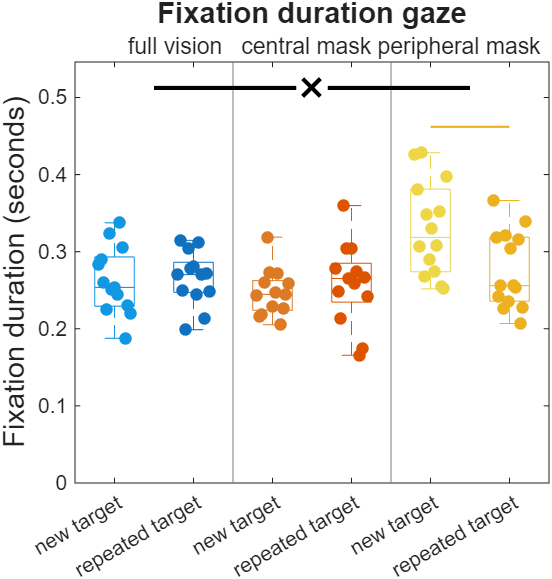


## Exploration phase fixation rate

### Lower frame rate Higher frame rate


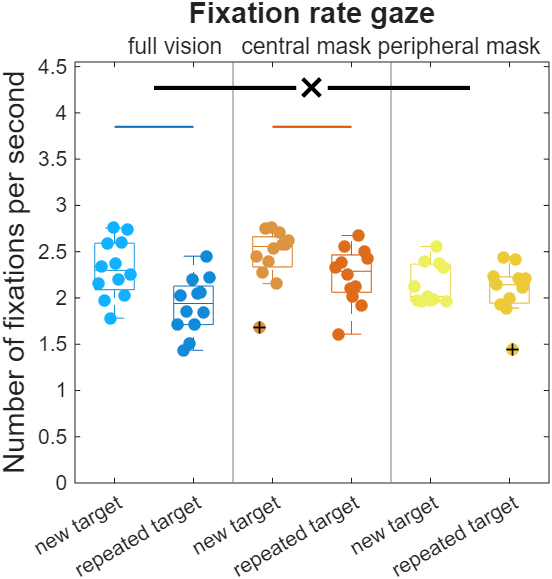

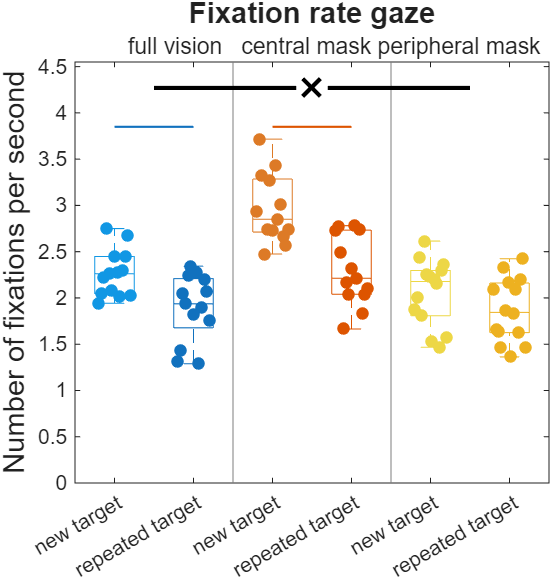


## Homing in phase duration

### Lower frame rate Higher frame rate


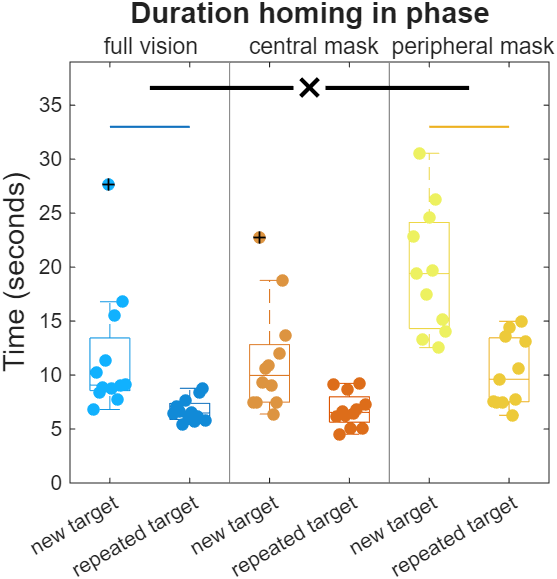

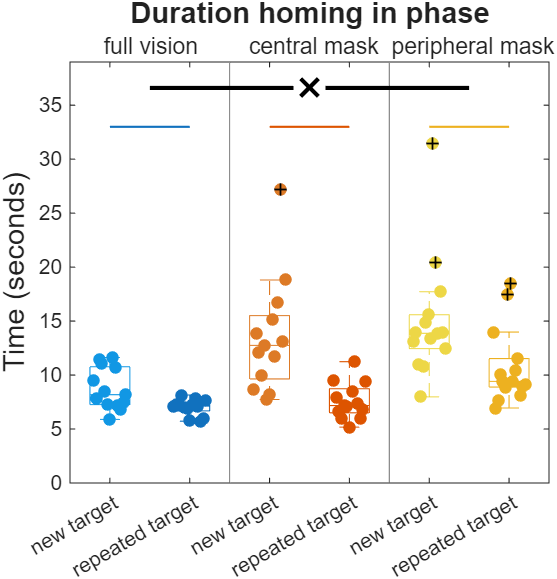

Supplement: Supplement 2 [file tvst-15-4-10_s002.docx]
